# Deruxtecan-Induced Reversible Fanconi Syndrome

**DOI:** 10.7759/cureus.40890

**Published:** 2023-06-24

**Authors:** Pooja Kalantri, Koba Lomashvili

**Affiliations:** 1 General Nephrology, Emory University, Atlanta, USA

**Keywords:** medication side effects, electrolyte abnormalities, onconephrology, trastuzumab-deruxtecan, drug induced fanconi syndrome

## Abstract

Increasingly complex and constantly emerging cancer treatment protocols are associated with kidney toxicities. Data clearly demonstrate that when patients with cancer develop acute or chronic kidney disease, severe fluid and electrolyte abnormalities, outcomes are inferior, and the promise of curative therapeutic regimens is lessened. We present a case of a 74-year-old woman with metastatic, recurrent ER+/PR-/HER2+ invasive ductal carcinoma of the right breast, status post bilateral mastectomies, chemotherapy, radiation therapy, and hormonal therapies, who were clinically stable on Trastuzumab/Pertuzumab maintenance for about a year. She then experienced disease progression. She was started on Trastuzumab+Deruxtecan (T-Dxt). However, due to worsening diarrhea of more than 12 episodes per day, decreased oral intake, weakness and weight loss, she got admitted to the hospital. Laboratory data showed hyponatremia, hypokalemia, non-anion gap metabolic acidosis, hypomagnesemia, and hypophosphatemia. These laboratory abnormalities were initially attributed to diarrhea. Renal losses were suspected when the electrolyte abnormalities did not correct despite improving diarrhea. Urine electrolytes were hence tested. There was evidence of Fanconi syndrome with glucosuria, proteinuria, and renal potassium and phosphorus wasting. Fanconi syndrome was attributed to the Deruxtecan component of the combination chemotherapy, as she was previously on Trastuzumab with no such abnormalities. The electrolyte abnormalities resolved over the course of a few months. To our knowledge, this is the first case of Fanconi syndrome due to T-Dxt.

## Introduction

Kidney toxicities are associated with increasingly complex and constantly emerging cancer treatment protocols. They include complications related to acute or chronic kidney disease and severe fluid and electrolyte abnormalities. Such patients have poor outcomes and some of them do not even qualify for curative therapeutic regimens as a result. However, despite therapy, outcomes in these patients have been inferior [1]. Here we present a case of a new chemotherapeutic agent and the associated electrolyte abnormalities that it has caused.

This article was previously presented as a poster titled "Onconephrology, Rise of the Unknowns: Enhertu-Induced Fanconi Syndrome" at the 2022 American Society of Nephrology (ASN)-Kidney Week on November 4, 2022 held at Orlando, Florida.

## Case presentation

A 74-year-old woman with metastatic, recurrent ER+/PR-/HER2+ invasive ductal carcinoma of the right breast, status post bilateral mastectomies, chemotherapy, radiation, and hormonal therapies, who, while being on Trastuzumab/Pertuzumab maintenance for a year, experienced progression. She was started on Trastuzumab+Deruxtecan (T-Dxt). Due to more than 12 episodes of diarrhea a day, decreased oral intake and weight loss, she was admitted to the hospital. Her blood pressure was soft, in the 90s and 100s systolic. Lab work was as noted in Table 1. Of significance, sodium was 132 mmol/L, potassium 2.1 mmol/L, bicarbonate 19 mmol/L, anion gap 10, calcium 7.3 mg/dL, magnesium 1.2 mg/dL, and phosphorus 1.3 mg/dL. Electrolyte abnormalities persisted with improving diarrhea prompting testing for renal wasting. Urine was positive for protein (3+ on dipstick) and glucose, with a pH of 5. The fractional excretion of potassium was 23.7 and phosphorus was 26.9. This is noted in Table 2. There was hence evidence of Fanconi syndrome with glycosuria (with normoglycemia), proteinuria, and renal potassium and phosphorus wasting. The Fanconi syndrome was attributed to the Deruxtecan component of the combination chemotherapy, as she was previously on Trastuzumab with no such abnormalities. She was also on no other medications that could cause Fanconi syndrome. T-Dxt was stopped. She was given mineral supplementation for potassium, phosphorus, and bicarbonate with improvement in her functional status. Mineral supplementation was stopped with a resolution of the Fanconi syndrome after two months.

**Table 1:** Renal function panel.

Renal function panel​	Value​	Units​	Normal range​
Sodium​	132​	mmol/L​	136-145 mmol/L​
Potassium​	2.1​	mmol/L​	3.5-5.5 mmol/L​
Chloride	103	mmol/L​	98-107 mmol/L​
Carbon dioxide	19	mmol/L	23-29 mmol/L
Anion gap​	10	--​	8-11​
Corrected calcium​	7.3​	mg/dL​	8.6-10.3 mg/dL​
Magnesium​	1.2​	mg/dL​	1.9-2.7 mg/dL​
Phosphorus​	1.3​	mg/dL​	2.5-5 mg/dL​
Blood urea nitrogen​	16​	mg/dL​	7-25 mg/dL​
Creatinine​	0.94​	mg/dL​	0.6-1.2 mg/dL​
Blood sugar​	96​	mg/dL​	70-105 mg/dL​

**Table 2:** Urine studies and values to calculate fractional excretions of potassium and phosphorus.

Parameter​	Value​
Urine glucose​	35 mg/dL​
Urine potassium​	29.2 mmol/L​
Urine phosphorus​	4.42 mmol/L​
Urine creatinine​	42.4 mg/dL​
Serum potassium​	3.6 mmol/L​
Serum phosphorus​	1.5 mg/dL or 0.48 mmol/L​
Serum creatinine​	1.24 mg/dL​

## Discussion

Fanconi syndrome is a defect of the proximal tubule leading to malabsorption of various electrolytes and substances that are usually absorbed by the proximal tubule leading to amino aciduria, low molecular weight proteinuria, hypokalemia, hypophosphatemia, metabolic acidosis (type 2 renal tubular acidosis), hypouricosemia, and glycosuria (even with normoglycemia) [2-3]. Possible mechanisms include widespread abnormality of most or all of the proximal tubule carriers, “leaky” brush border or basolateral cell membrane, inhibited or abnormal Na+/K+ ATPase pump, impaired mitochondrial energy generation, or another cell organelle dysfunction [4]. It can be an inherited or an acquired condition. While inborn errors of metabolism are the common cause in children, in adults, it has been associated with exogenous toxins [l-lysine and l-arginine, aristolochic acid (Chinese herb nephropathy), fumaric acid, suramin, paraquat], heavy metals (lead, cadmium, mercury, copper), and various medications as mentioned below [4-5]. The most serious complications are bone demineralization from phosphaturia and progressive chronic kidney disease [3].

Drug-induced Fanconi syndrome has been associated with nucleotide reverse-transcriptase inhibitors (tenofovir, adefovir, didanosine, lamivudine, stavudine), anticancer drugs (ifosfamide, oxaliplatin, cisplatin), anti-convulsant drugs (valproic acid, topiramate), antibiotics (aminoglycosides, expired tetracyclines), DNA polymerase inhibitor (cidofovir), deferasirox, streptozocin, lenalidomide, apremilast [5]. Some of these are reversible, while others are not.

We have a case of reversible Fanconi syndrome due to T-Dxt.

In DESTINY-Breast04 clinical trial involving patients with HER2-low metastatic breast cancer, Enhertu (T-Dxt) resulted in significantly longer progression-free and overall survival than the physician's choice of chemotherapy (Funded by Daiichi Sankyo and AstraZeneca; DESTINY-Breast04 ClinicalTrials.gov number, NCT03734029). Based on the results of the DESTINY-Breast04 trial, as of August 5, 2022, it has been FDA-approved for adult patients with unresectable or metastatic HER2-low breast cancer who have received prior chemotherapy in the metastatic setting or developed disease recurrence during or within six months of completing adjuvant chemotherapy (fda.gov website).

Serious adverse reactions occurred in 28% of patients receiving Enhertu [6]. These were interstitial lung diseases/pneumonitis, pneumonia, dyspnea, musculoskeletal pain, sepsis, anemia, febrile neutropenia, hypercalcemia, nausea, pyrexia, and vomiting (fda.gov website) [6]. The most common (≥20%) adverse reactions, including laboratory abnormalities, were nausea (76%), decreased white blood cell count (70%), decreased hemoglobin (64%), decreased neutrophil count (64%), decreased lymphocyte count (55%), fatigue (54%), decreased platelet count (44%), alopecia (40%), vomiting (40%), increased aspartate aminotransferase (38%), increased alanine aminotransferase (36%), constipation (34%), increased blood alkaline phosphatase (34%), decreased appetite (32%), musculoskeletal pain (32%), diarrhea (27%), and hypokalemia (25%) [6].

There has never been a mention of Fanconi syndrome so far in the literature to our knowledge due to T-Dxt. The mechanism of action of T-Dxt is as follows: following binding to HER2 on tumor cells, T-Dxt undergoes internalization and intracellular linker cleavage by lysosomal enzymes. Upon release, the membrane-permeable Dxt enters the nucleus and causes DNA damage and apoptotic cell death [7]. We think that Dxt also affects the proximal tubular cells in a similar mechanism, and this is possibly leading to Fanconi syndrome. 

As seen in our patient, it looks like she developed Fanconi syndrome 1-2 weeks after starting treatment, and was resolved in about 2-3 months after the last dose. Interestingly enough, the patient continued having mild hypomagnesemia (1.6-1.8 mmol/L), which was present even before the T-Dxt treatment and persisted intermittently after discontinuation of the treatment. This was thought to be due to intermittent gastrointestinal upset. The hypocalcemia did resolve completely though.

## Conclusions

We live in an exciting era of cell-specific anticancer therapies. New biochemical pathways and agents modulating them are constantly identified. Some newly approved therapies can result in severe side effects, causing significant morbidity and precluding further treatment. It is imperative to recognize and treat complications early to allow for successful treatment. We report the first case (per our knowledge) of reversible Fanconi syndrome due to Trastuzumab/Deruxtecan and her case illustrates the importance of early diagnosis and treatment of drug-induced Fanconi syndrome. It is also something that primary care physicians, hospitalists, oncologists, and nephrologists should keep an open mind about when presented with a similar scenario.
